# Virtual Peer Mentoring for Adolescents With Congenital Heart Disease: A Mixed-Methods Study of the iPeer2Peer Program in the Transition to Adult Care

**DOI:** 10.1016/j.cjcpc.2025.04.003

**Published:** 2025-04-17

**Authors:** Tieghan Killackey, Sofia Olaizola, Fareha Nishat, Richard Xi, Navreet Gill, Sandra Aiello, Rafael Alonso-Gonzalez, Conall Morgan, Jennifer Graham, Laura Veloso, Cindy Desbiens, Charolette Desbiens, Jennifer N. Stinson, Sara Ahola Kohut

**Affiliations:** a Child Health Evaluative Sciences, The Hospital for Sick Children, Toronto, Ontario, Canada; b Peter Munk Cardiac Centre, University Health Network, Toronto, Ontario, Canada; c Lawrence Bloomberg Faculty of Nursing, University of Toronto, Toronto, Ontario, Canada; d Canadian Congenital Heart Alliance, Toronto, Ontario, Canada; e Labatt Family Heart Centre, The Hospital for Sick Children, Toronto, Ontario, Canada

## Abstract

**Background:**

Adolescents with congenital heart disease (AWCHD) experience various symptoms, which can restrict social interactions and negatively impact quality of life. iPeer2Peer is an evidence-based virtual peer mentorship program that has improved outcomes for youth in several chronic illness populations. This study sought to examine the feasibility and acceptability of delivering the iPeer2Peer program to AWCHD.

**Methods:**

A convergent, parallel mixed methods pre-post study design was used. AWCHD (13-18 years old) were recruited and matched with trained peer mentors (18-25 years old). Matched dyads completed up to 10 video calls over 15 weeks. Primary outcomes focused on engagement and acceptability (ie, accrual, withdrawal, number of calls, and qualitative feedback). Secondary outcomes focused on estimates of program effectiveness for this population (ie, transition readiness, quality of life, and self-efficacy).

**Results:**

Study results demonstrated a participant accrual rate of 25% (19 of 76) and an enrollment rate of 95% (18 of 19). Eighteen adolescents (mean age 16.5 years, 50% female) were enrolled with a range of CHD diagnoses. A total of 16 mentees completed the program, with 2 lost to follow-up. Adherence to the study protocol was strong, with 74% of mentees completing study measures at the end of the program (T2) and at 6 months after the program (T3). The average number of calls per pair was 7.25, and the average call length was 40.25 minutes. Transition readiness and self-management increased across program time points (with statistically significant improvement from baseline to 6-month follow-up) and aligned with qualitative results that illustrated the benefits of social support in the transition to adult care.

**Conclusions:**

This study demonstrated that the iPeer2Peer program was acceptable in this population. AWCHD were highly engaged and described the benefits of peer support during the transition process. Clinical programs may consider implementing similarly structured peer mentorship initiatives as a unique form of psychosocial support during the transition adult care.

## Résumé

**Contexte:**

Les adolescents atteints d’une maladie cardiaque congénitale (MCC) présentent tout un éventail de symptômes qui peuvent restreindre leurs interactions sociales et nuire à leur qualité de vie. Le programme de mentorat virtuel iPeer2Peer axé sur des données probantes a eu des effets positifs chez les jeunes appartenant à plusieurs populations atteintes de maladies chroniques. Cette étude visait donc à examiner la faisabilité et l’acceptabilité du programme iPeer2Peer pour les adolescents atteints d’une MCC.

**Méthodologie:**

Nous avons réalisé une étude pré-post à méthodes mixtes, convergente et parallèle. Nous avons recruté des adolescents (âgés de 13 à 18 ans) atteints d’une MCC, que nous avons appariés à des mentors (âgés de 18 à 25 ans) ayant reçu une formation. Les dyades appariées ont effectué jusqu’à 10 appels vidéo en 15 semaines. Les principaux paramètres évalués portaient sur le niveau de participation et l’acceptabilité (c.-à-d. le taux d’inclusion, les abandons, le nombre d’appels et les rétroactions qualitatives). Les paramètres secondaires visaient à estimer l’efficacité du programme dans cette population (c.-à-d. préparation à la transition, qualité de vie et auto-efficacité).

**Résultats:**

Les résultats de l’étude indiquent un taux de consentement de 25 % (19 sujets sur 76) et un taux réel d’inclusion de 95 % (18 sujets sur 19). Dix-huit adolescents atteints de tout un éventail de MCC (âge moyen : 16,5 ans; 50 % de sujets de sexe féminin) ont donc participé au programme. Un total de 16 mentorés ont terminé le programme; deux ont été perdus de vue. Une forte observance du protocole de l’étude a été constatée : 74 % des mentorés avaient terminé les évaluations de l’étude à la fin du programme (T2) et 6 mois après le programme (T3). Le nombre moyen d’appels par paire a été de 7,25, et la durée moyenne d’un appel était de 40,25 minutes. La préparation à la transition et la capacité d’autoprise en charge se sont améliorées au fil du programme (amélioration statistiquement significative après 6 mois de suivi par rapport au début de l’étude), et les résultats cadrent avec les données qualitatives ayant mis en évidence les bienfaits associés au soutien social lors de la transition vers les soins pour adultes.

**Conclusions:**

Cette étude montre que le programme iPeer2Peer est acceptable dans cette population. Les adolescents atteints d’une MCC ont manifesté un fort niveau de participation et ont indiqué que le soutien des pairs leur avait été bénéfique durant le processus de transition. Il faut envisager de mettre en œuvre de telles initiatives structurées de mentorat par les pairs dans le cadre des programmes cliniques pour offrir cette forme particulière de soutien psychosocial pendant la transition vers les soins pour adultes.

Throughout the lifespan, patients living with congenital heart disease (CHD) require ongoing treatment and follow-up to manage symptoms and ensure rapid identification of changes in cardiovascular status. Specifically, adolescents with CHD (AWCHD) can experience many physical (eg, shortness of breath, fatigue, and pain) and psychological (eg, anxiety, depression, and cognitive impairment) symptoms that can be distressing and may restrict physical and social interactions, ultimately challenging adolescent development and negatively impacting health-related quality of life (HRQL).^1^, ^2^, ^3^, ^4^, ^5^ During this life stage, AWCHD are encouraged to develop a sense of independence, manage adverse effects, and plan for the transition to adult care.^6^ This period can be challenging for all AWCHD, but especially for those with complex CHD, who have an increased risk of cognitive deficits (ie, inattention and challenges with executive function), which can significantly influence transition readiness and negatively impact transition outcomes.^7^ Late adolescence and early adulthood is a time of significant risk for patients to have lapses in care, and approximately one-quarter of young adults fail to attend an adult CHD clinic once they have aged out of pediatric care.^8^, ^9^, ^10^ Patients experiencing lapses in care have an increased need for urgent intervention and have an increased risk of hospital admission.^11^^,^^12^ Accordingly, interventions designed to support coping and CHD self-management represent key strategies to improve the ability of AWCHD to manage the multiple ways that heart disease impacts their lives and successfully transition to adult care.^6^^,^^7^^,^^11^^,^^13^^,^^14^

Social support has also been shown to improve patient health outcomes by promoting disease self-management, improving quality of life, and decreasing anxiety and depression in adult heart disease populations.^14^, ^15^, ^16^, ^17^, ^18^ The presence of strong social support, along with knowledge and understanding of their cardiac abnormality, has also been significantly associated with improved HRQL in AWCHD.^19^^,^^20^ A review of peer support interventions for adolescents with chronic illnesses demonstrated that peer support improves adolescents’ behavioral and emotional symptoms; however, none of the included studies targeted the CHD population.^21^ A recent randomized controlled trial (RCT) investigated a multicomponent transition program for AWCHD that included a one-time peer support component and found significant increases in empowerment and disease-related knowledge after the program.^22^ Another recent systematic review investigating strategies to promote successful transitions to adult care found that AWCHD are widely interested in mobile-based programs, recommending future work to investigate a virtual peer mentorship program.^23^

The iPeer2Peer program is an evidence-based virtual peer support program that provides an opportunity for adolescents with chronic illnesses to connect with young adults with similar experiences through video calling platforms. Peer mentors are nominated by their health care teams and trained with an established framework. The program has been tested in 6 adolescent populations, including those with chronic pain, juvenile idiopathic arthritis, and cancer, and has demonstrated success in improving disease self-management and coping efficacy.^24^, ^25^, ^26^

With the success of the iPeer2Peer program in other adolescent populations with chronic illnesses, this study sought to explore the opportunity to deliver this program to a population of AWCHD. The primary objectives of this study were (1) to determine whether the iPeer2Peer program is feasible and acceptable for the AWCHD population and could be implemented into clinical practice, and (2) to examine the impact of the iPeer2Peer program on self-report measures of transition readiness, HRQL, cardiac knowledge, social support, and self-efficacy in their ability to independently manage CHD-related symptoms immediately and 6 months after the program.

## Methods

### Study design

Using a convergent, parallel mixed methods pretest posttest study design, we evaluated the acceptability and feasibility of the iPeer2Peer program for AWCHD and examined the program’s effectiveness in this clinical population.^27^ Our qualitative and quantitative data were collected concurrently and analyzed separately, and then integrated to provide insight into the acceptability of the program.^28^

The iPeer2Peer program is a virtual mentorship program that gives the opportunity for adolescents living with a chronic condition to connect with young adults with the same chronic condition. Throughout the program, adolescents can learn from young adult mentors who have successfully transitioned to adult care and learned to manage their condition. Participants use video calling platforms (ie, Skype, Zoom, Facetime, and WhatsApp) to complete up to 10 calls over the course of 15 weeks and are required to communicate and schedule calls based on their availability and needs. The program’s success relies on collaboration with clinic staff to nominate potential mentors and identify mentees who would benefit.

### Setting

The SickKids Labatt Family Heart Centre and the University Health Network Peter Munk Cardiac Centre collaboratively launched the CHD transition program (CHDTP) in January 2020. The program supports transition to adult care, by providing nurse-led, 1:1 tailored education sessions for AWCHD. This program addressed a growing need by providing continuity of care for this population of youth. Research ethics board approval was obtained for this study from the Hospital for Sick Children (REB #1000077477).

### Participants

#### Mentee participants

AWCHD were recruited from the Hospital for Sick Children between June 2022 and October 2023 from the CHDTP. Inclusion criteria for adolescents included (1) English-speaking, (2) 14-18 years old with capacity to give informed consent, (3) CHD diagnosis with an expected 5-year survival rate >70% according to cardiologist, and (4) access to a device capable of using free video calling software (ie, Skype, WhatsApp, and FaceTime). Loaner devices were available to participants if needed to complete the program. Participants were excluded if they (1) had significant cognitive impairments or major comorbid illness (ie, psychosis and suicidal ideation), (2) received surgery for CHD diagnosis in the last 6 months, (3) were participating in other peer support programs, and (4) were receiving end-of-life care. Exclusion criteria were verified with the clinic team.

Eligible patients were identified via initial chart review by research staff or nominated by their health care provider (HCP). Informed consent was obtained remotely through Research Electronic Data Capture (REDCap). Baseline questionnaires were administered to obtain self-reported demographic and disease-related information including gender, age, ethnicity, diagnosis, medications, and surgical history, using an investigator-developed tool. On baseline questionnaire completion, mentees were matched with a peer mentor. Pairings were determined by HCP recommendations based on diagnosis and disease severity, common interests, and gender preferences (if relevant).

#### Mentor participants

Recruited peer mentors were young adults living with CHD who successfully transitioned to adult care and learned to self-manage their condition. Mentors were identified and nominated by their HCP team. Inclusion criteria for young adult mentors included (1) English speaking 18- to 25-year-old adults living with CHD, (2) not currently undergoing any major cardiac interventions or surgeries, (3) have had at least 1 appointment at the adult CHD clinic at Toronto General Hospital (4) nominated by care team/co-investigators as a good candidate, (5) self-reported adherence to treatment plan and ability to manage CHD-related symptoms, and (6) access to a device capable of using free video calling software. Mentors were excluded if they were diagnosed with an untreated major psychological illness.

**Figure 1:**
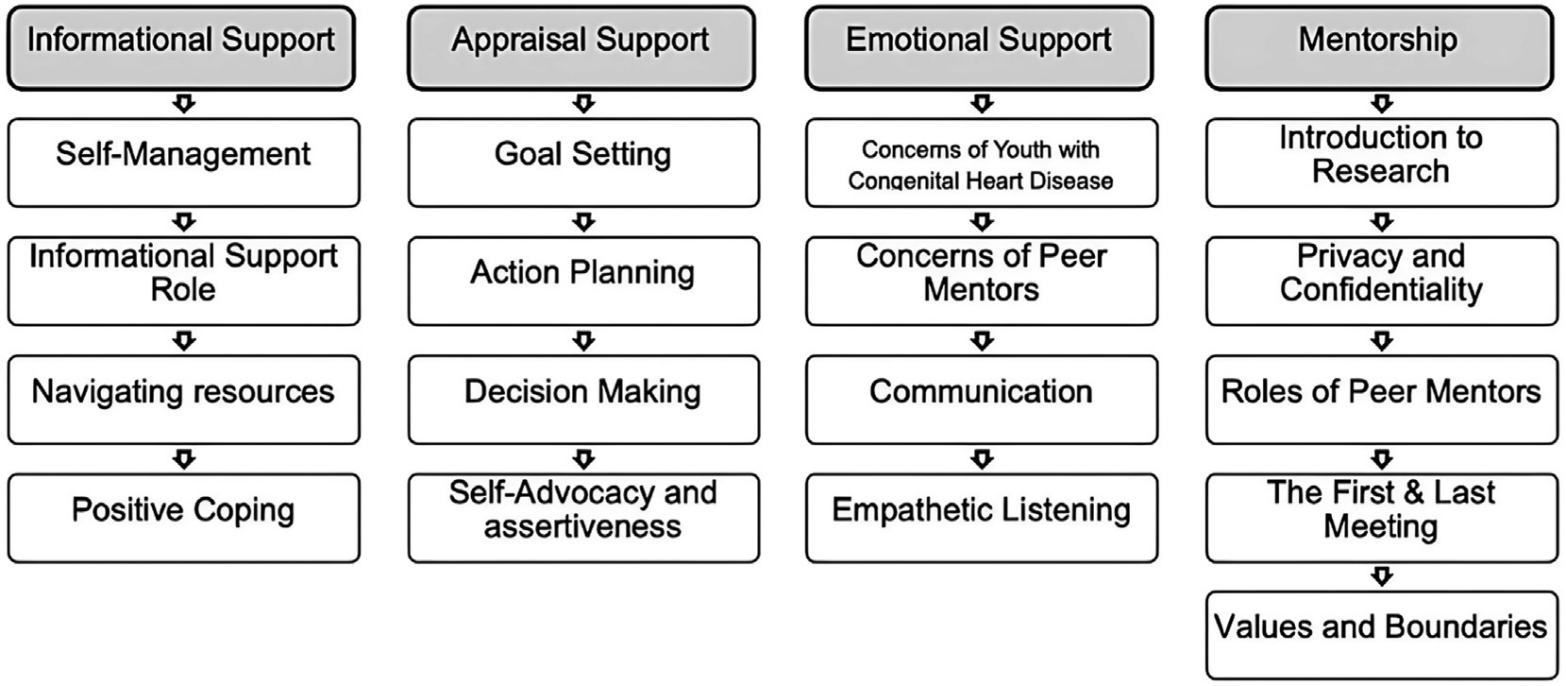
Peer mentor training course outline.

On enrollment, mentors completed self-report baseline measures including demographic (ie, gender, age, and ethnicity, using an investigator-developed demographic tool) and disease-related information (ie, diagnosis, medications, and surgical history by participant self-report). All recruited mentors completed the same manualized, validated 2-day virtual training sessions (total of 20 hours). Training involved a stepwise approach comprising lectures, active group discussions, case examples, small group activities, and role-play activities. Topics covered throughout the training sessions are listed below (see Fig. 1). Peer mentors could reach out to the research team for support or additional training materials and resources if needed. Throughout the program, mentor responsibilities included recording mentorship calls and sending audio files to research staff for security reasons, completing call logs via REDCap to document conversation topics (ie, CHD education-related, general, etc). Mentors received remuneration throughout the program for their time including for mentor training and completing calls with their mentees.

#### Health care provider participants

Eligible HCPs were recruited through a study invitation letter sent via email. Consented HCPs provided feedback on acceptability and satisfaction through quarterly surveys and participation in a focus group discussion.

### Outcomes

#### Primary outcomes

##### Feasibility

1. *Participant accrual.* Accrual rates were calculated from study tracking logs. Accrual was calculated as those consented over those approached and those enrolled over those consented.
2. *Withdrawal rate*. Withdrawal rates were calculated from study tracking logs. Withdrawal was calculated as those lost to follow-up (ie, those who did not complete the study questionnaire at T3) over those completed.
3. *Adherence to study protocol.* Adherence rates were calculated from REDCap logs as those who completed each questionnaire over those enrolled in the study, at each time point.

##### Engagement

Engagement outcomes were assessed via number of calls completed and length of calls. Mentors sent audio recordings of completed calls to research staff via SickKids Secure File Transfer. Research staff kept a call log to report the number of calls completed per dyad pair and the length of each call. Mentors also completed call log surveys on REDCap to report the date of call, outcome of call, and topics discussed. Using evidence from previous iPeer2Peer trials, engagement is defined by 3 categories: low engagement is 1-3 calls, moderate engagement is 4-6 calls, and high engagement is 7+ calls.^29^

##### Acceptability

Acceptability of and satisfaction with the program were evaluated through semistructured phone interviews with mentees and mentors. A focus group was held with HCPs at the half-way point and end point of the study to explore acceptability of and satisfaction with the program, along with considerations of implementation and potential for long-term sustainability within routine clinical care.

##### Adverse events

Unanticipated problems and adverse events were tracked and reported from study tracking logs.

#### Secondary outcomes

##### Mentees

Outcomes were assessed at 3 time points: T1 (baseline measures), T2 (immediately after the program), and T3 (6 months after the program). Effectiveness of the iPeer2Peer program was evaluated by self-management and transition readiness (TRANSITION-Q^30^), HRQL (PedsQL Cardiac Module^31^), knowledge (SickKids CHD Knowledge Questionnaire), perceived social support (PROMIS Pediatric Peer Relationship Scale^32^), self-efficacy (Generalized Self-Efficacy Sherer Scale^33^), mentor quality (Mentor Behavior Scale^34^), and mentor support (Peer Mentor Support Scale^35^).

##### Mentors

Measures of physical and emotional symptoms (SF-36^36^) and role satisfaction (PROMIS Satisfaction with Social Roles and Activities^37^) were completed before peer mentor training and at study completion. See Table 1 for descriptions of each tool.

**Table 1:** Description of secondary effectiveness measures

Outcome	Measurement tool	Description
Mentee measures		
Self-management	TRANSITION-Q^30^	A 14-item generic tool to capture self-management skills in adolescents (12-18 year olds) with chronic conditions. Response options are 2 (“always”), 1 (“sometimes”), or 0 (“never”). Raw scores (range 0-28) are transformed to a 0-100 scale, with higher scores indicating greater self-management.
HRQL	PedsQL Cardiac Module^31^	A 27-item multidimensional scale (heart problems and treatment, treatment II, perceived physical appearance, treatment anxiety, cognitive problems and communication), rated using a 5-point Likert scale ranging from 0 (“never a problem”) to 4 (“almost always a problems”). Items are reverse scored and linearly transformed to a 0-100 scale, with higher scores indicating better HRQOL.
Self-efficacy	Investigator modified version of the Generalized Self-Efficacy Sherer Scale^33^	A 12-item scale used to assess self-efficacy, rated using a 5-point Likert scale ranging from 1 (strongly disagree) to 5 (strongly agree). A total summed score was calculated, with higher scores indicating less self-efficacy.
Perceived social support	PROMIS Pediatric Peer Relationship Scale—Short Form^32^	An 8-item scale used to measure the quality of self-reported peer relationships. Items are rated using a 5-point Likert scale ranging from 1 (“never*”*) to 5 (“almost always”). Total raw scores are transformed to a standardized T-score (population mean = 50, standard deviation = 10), with higher scores suggesting better peer relationships.
Disease knowledge	SickKids CHD Knowledge Questionnaire	A 14-item scale, developed by the SickKids Cardiac Transition Program, used to measure CHD-specific knowledge. Response options ranged from 1 (“I have no idea”) to 5 (“I know all about this [I'm there!]”). A total summed score was calculated, with higher scores suggesting greater CHD knowledge.
Mentor quality	Mentor Behavior Scale^34^	A 15-item multidimensional (structure, engagement, autonomy support, and competence support) scale measuring mentor's role in providing guidance, emotional support, and autonomy to mentees. Items are rated on a 5-point Likert scale from 1 (“does not apply at all”) to 5 (“applies very well”). A total summed score was calculated, with higher scores indicating stronger mentor support behaviors.
Mentor support	Peer Mentor Support Scale^35^	A 17-item scale used to measure the support (capturing informational, affirmational, and emotional aspect**)** provided by peer mentors, rated on a 4-point Likert scale ranging from 1 (“strongly disagree”) to 4 (“strongly agree”). A total summed score was calculated, with higher scores corresponding with greater peer-mentor support.
Mentor measures		
Physical and emotional symptoms	Short Form Health Survey-36^36^	A 36-item multidimensional scale (physical functioning, role limitations due to physical health, bodily pain, general health perceptions, vitality, social functioning, role limitations due to emotional health, and mental health) used to measure health-related quality of life. Total raw scores are transformed to 0-100 scale, with higher scores indicating better health status and quality of life.
Role satisfaction	PROMIS Satisfaction with Social Roles and Activities^37^	A 6-item scale used to measure satisfaction with social roles and activities. Items are rated using a 5-point Likert scale ranging from 1 (“not at all”) to 5 (“very much*”*). Total raw scores are transformed to a standardized T-score (population mean = 50, standard deviation = 10), with higher scores suggesting greater satisfaction with social roles and activities.

CHD, congenital heart disease; PedsQL, Pediatric Quality of Life Inventory; PROMIS, Patient-Reported Outcomes Measurement Information System.

### Data analysis

#### Quantitative analysis

For both mentors and mentees, demographic and disease characteristics were described with mean and standard deviation for continuous variables and raw counts and percentages. Linear mixed models were used to assess the effects of the iPeer2Peer program on secondary effectiveness outcomes in mentees. The model included a 3-level categorical variable for time point (with baseline as the reference) as the fixed effect and intercepts for each participant as random effects. A significance level of α = 0.05 was applied to self-management and transition readiness measured by the TRANSITION-Q. A Bonferroni-adjusted alpha level of α = 0.005 was used to maintain an overall level of 0.05 for all the remaining outcomes. *Post hoc* comparisons were conducted to investigate the change in scores between the end of the program (T2) and at 6 months after the program (T3) for all outcomes, with a Bonferroni-adjusted alpha applied to account for multiple comparisons. Model diagnostics were performed to assess assumptions of linearity and homoscedasticity. Linearity of residuals was assessed using Q-Q plots and histograms with normal overlays, whereas the residuals were plotted against fitted values to assess for homoscedasticity. Linearity assumption was met if the residuals followed a straight line in the Q-Q plot and a roughly symmetric distribution in the histogram was present. Homoscedasticity was met if the residuals showed a similar vertical spread across the fitted values. Secondary outcomes for mentors were described using mean and standard deviation (for normally distributed outcomes) or median and interquartile range (for non-normally distributed outcomes) at baseline and the end of the program (T2). Data were analyzed using STATA version 15.1 (StataCorp LLC, College Station,Texas).

### Qualitative analysis

Qualitative interviews with mentees, mentors, and HCPs were audio-recorded, transcribed, and analyzed using a combination of inductive and deductive qualitative content analysis.^38^ Codes were generated inductively directly from the data using the language of participants, in combination with deductive use of key concepts from previous reports of the iPeer2Peer program.^38^ Coding of transcripts took place on Dedoose software, a cross-platform app for analyzing qualitative and mixed methods data.^39^ An initial coding framework was developed by 2 authors (SO and RX); after the initial codebook was established, 3 team members coded transcripts from each participant group (mentee, mentor, and HCP). Codes were compared and modified, and overarching categories were developed to group the codes. Once all transcripts had been coded once, 2 authors (SO and RX) reviewed all previously coded transcripts with the finalized codebook to ensure that all codes were captured. A team of coauthors (TK, SO, RX, and JS) reviewed the categories and contributed to the development of category names and descriptions. Quotations from a range of participants were included to ensure that a variety of perspectives were represented across each category. Finally, significant findings from both the quantitative and qualitative analyses were combined to highlight key results related to transition readiness and program acceptability.

## Results

### Primary outcomes

#### Feasibility

##### Participant accrual

**Figure 2:**
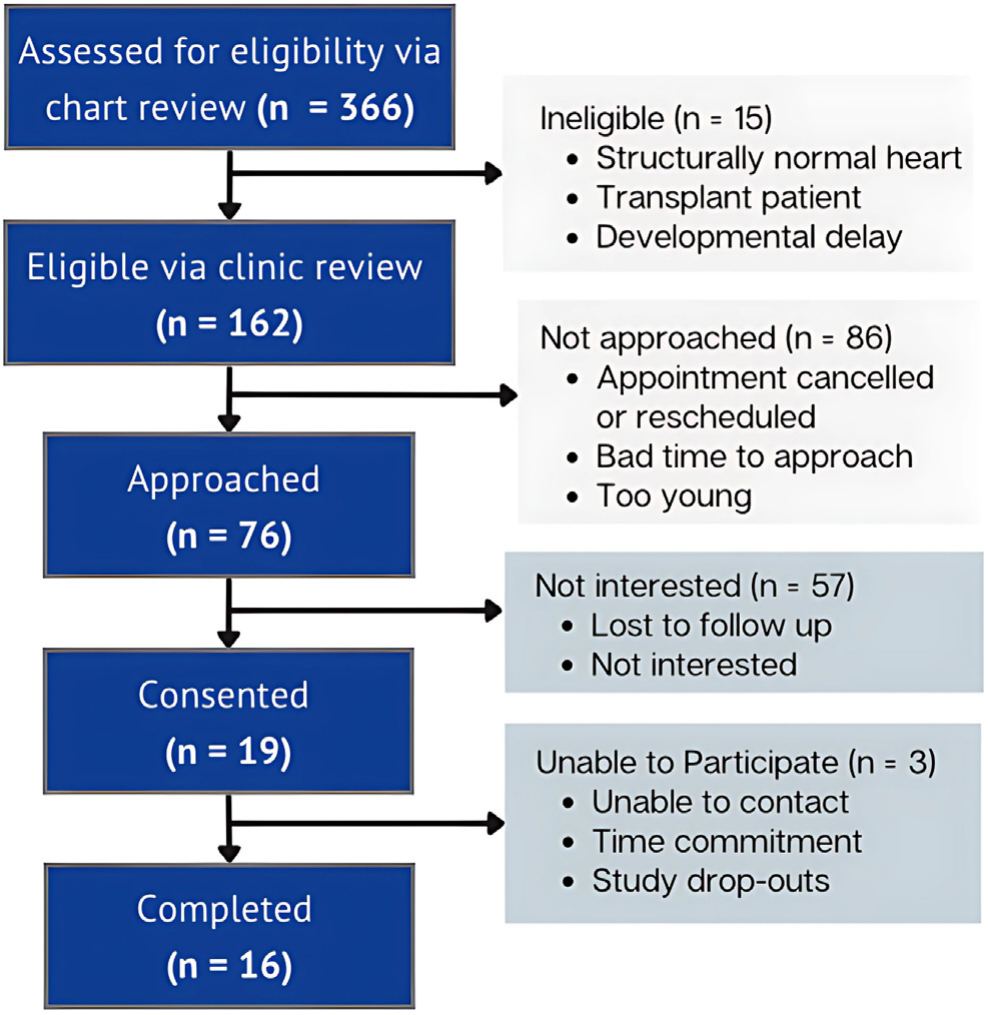
Consort study flow diagram.

Participant enrollment began in June 2022 and ended October 2023, with final follow-up completed in September 2024. The Consolidated Standards of Reporting Trials (CONSORT) is reported below (Fig. 2). Across the recruitment period, 19 mentee participants consented to participate in the iPeer2Peer program. Although there were a large number of eligible patients, the majority were not interested in participating in the study for a variety of reasons (eg, time commitment and not yet preparing for transition). The actual percentage of participants consented over those approached was 25% (19 of 76), and the percentage of participants enrolled over consented was 95% (18 of 19).

Participant demographics and baseline characteristics are presented in Table 2. There was an even split of participants by gender, and the average age of mentees was 16.5 (standard deviation 0.75) years. Mentees had a range of congenital heart defects from simple to complex, with the majority of participants (57%) having complex CHD (see Table 2 for an overview of CHD classifications).

**Table 2:** Baseline characteristics of mentees and mentors

Characteristics	Total
Mentee characteristics (n = 18)	
Age (y), mean (SD); range	16.5 (0.75); 15-17
Sex, n (%)	
Female	9 (50)
Male	9 (50)
Gender, n (%)	
Women	9 (50)
Men	9 (50)
Ethnicity, n (%)	
North American	4 (22)
Caribbean	2 (11)
European	4 (22)
West Asian/Middle Eastern	1 (6)
East Asian	1 (6)
South or Southeast Asian	2 (11)
Multiple ethnicities	4 (22)
Disease classification∗, n (%)	
Simple	1 (6)
Moderate	7 (37)
Complex	10 (53)
Mentor characteristics (n = 7)	
Age (y), mean (SD); range	21.7 (1.80); 19-24
Sex, n (%)	
Female	3 (43)
Male	4 (57)
Gender, n (%)	
Women	3 (43)
Men	4 (57)
Ethnicity, n (%)	
European	2 (29)
South or Southeast Asian	3 (43)
Multiple ethnicities	2 (29)
Disease classification∗, n (%)	
Simple	0 (0)
Moderate	3 (43)
Complex	4 (47)

SD, standard deviation.

∗ Classifications are based on participant self-report of diagnosis using the 2018 American Heart Association/American College of Cardiology Guideline for the Management of Adults With Congenital Heart Disease.^40^

The average mentor age was 22.2 years (19.8-24 years) with relatively even split between genders as well as a range in severity of CHD diagnoses (53% complex CHD). The average number of mentees that a mentor was paired with throughout the program was 3.4 (1-5).

##### Withdrawal rate

Of the 19 consented mentee participants, 16 completed the program, 1 dropped out, and 2 were lost to follow-up. The withdrawal rate at baseline (T1) was 5% (1 of 19). At the end of the program (T2) and at 6 months after the program (T3), the withdrawal rate was 26% (5 of 19). Nine mentors were enrolled into the program with 2 mentors dropping out at the start of the program. Of the 7 remaining mentors, 5 were active in the program completing mentorship calls with mentees.

##### Adherence

Adherence to the study protocol was strong; all mentees completed baseline questionnaires, with 74% completing them at the end of the program (T2) and at 6 months after the program (T3). Similarly, all mentors completed questionnaires at baseline, with 72% (5 of 7) completing them at the end of the program (T2). See Supplemental Table S1 for details.

##### Engagement

Throughout the study, 16 dyads completed the program, totaling 117 calls. The average number of calls completed per dyad was 7.25 (1-12), and the average call length in minutes was 40.25 (21-71).

#### Acceptability

##### Program satisfaction

Analysis of 14 semistructured interviews with mentees and mentors demonstrated that participants reported high satisfaction and acceptability with the program, praising mentor training and their positive call experiences. Mentees valued the practical advice and emotional support, rating their engagement an average of 8.4 on a scale of 1-10.

Specifically, the program fostered a strong sense of social support among participants, enhancing their mental and emotional well-being, and allowing adolescents to connect with others who shared similar experiences.

The mentor had the same heart defect that I do. So it’s just kind of good knowing there are other people like me out there. (17-year-old man mentee)

I was able to support them and help them feel like they weren’t alone and with their congenital heart defects, because many of them didn't know anyone in their schools or any of their friends who had congenital heart defects. (24-year-old woman mentor)

Further, mentor-mentee interactions frequently addressed broader subjects such as navigating the health care system, managing personal health challenges (ie, experiences with past hospitalizations and procedures), and discussing personal interests (ie, hobbies, pets, travel, work, and relationships). These conversations fostered strong interpersonal connections between participants. In fact, mentors found the interactions mutually rewarding, describing the experience as fulfilling and allowing them to use their own lived experiences and reflect on their own journeys:

For sure I definitely had some changes too... as a few calls went on, […] I was able to figure out a lot of things with myself... it puts a lot of perspective on what this generation has been going through and that's really opened my eyes to seeing a world in a completely different perspective. I found some kind of growth of just learning a lot of new experiences from other people. (22-year-old woman mentor)

Both mentees and mentors provided feedback for program improvements, identifying specific areas for development. One key suggestion was the inclusion of more resources addressing body image concerns, like managing scars from medical procedures:

There wasn’t really a section on like, self-image... when you go through procedures, you have scars. And I know that was a big discussion I had with many of my mentees... scars on our chest or from catheterizations. That could be something to consider. (24-year-old woman mentor)

##### Implementation and sustainability

In a focus group with HCPs, positive aspects of the program were highlighted including its accessibility and appeal to teens, being a virtual program:

The virtual piece is obviously a positive thing as well.... Having the opportunity to connect virtually [...] makes it easier for them and more convenient. (nurse 1, CHDTP)

HCPs provided suggestions for future program implementation, including opening the program to young adults who are in the early stages of their transition to adult care. This would allow patients who struggled with the transition to participate and provides additional opportunities to those who may not have been interested before they transitioned out of the pediatric setting:

That could be really helpful for those patients who don’t have a positive transfer [...] they probably might be interested in reaching out to some older patients with congenital heart disease. (nurse 1, CHDTP)

HCPs also advocated for parent targeted interventions and placed emphasis on the benefit for families to meet other parents or caregivers who have been through the transition with their own teens. They reported seeing parents also struggle with the transition process and highlighted that parents may need support in easing the anxiety of letting go of medical responsibility for their teen:

I feel like family members would really benefit from it, like I know a lot of families struggled and parents [are] often very anxious during the transition period. (nurse 2, CHDTP)

Importantly, HCPs provided feedback on the most appropriate age groups the program should target to provide maximum benefit. According to HCPs, patients younger than 16-17 years were not yet engaged in transition planning, which led to limited interest in the peer support program. Throughout recruitment, feedback from nurses noted that in suggesting the program to AWCHD younger than 16 years, the patients were often disinterested with nurses indicating that they were “too young” for the program. In addition, HCPs suggested that adolescents with more complex forms of CHD stand to benefit more from the program than those with simpler cases:

I think perhaps this program might be better suited for teens with more severe congenital heart disease versus those who have [...] simpler forms of it just because those that are more complex seem to be in the system more often and seem to perhaps struggle more with their mental health. (nurse 2, CHDTP)

HCPs discussed the sustainability of the program outside of a research context. “Pitching” the program to patients became a routine for them and was feasible to incorporate in their clinic visits. However, they noted that the program being offered in a research setting was also barrier for teens due to the need to fill out forms and questionnaires at multiple time points. In addition, in discussing the sustainability of the program within their clinic, HCPs emphasized that the program requires dedicated personnel and time to maintain mentor engagement and facilitate strong dyad matches:

Somebody’s going to have to own it for it to be sustainable so resources it’s going to it’s fall into the hands of the available resources, which is always the tricky piece[...] It would need to have somebody to have some dedicated time to make it sustainable. (nurse 1, CHDTP)

Ultimately, HCPs were highly enthusiastic about the program as it addressed an existing need for psychosocial support within this unique clinical population.

### Secondary outcomes

#### Program effectiveness

Results from the linear mixed model suggest that on average self-management and transition readiness increased (β = 6.37, 95% confidence interval: 1.98-10.7, *P* = 0.005) from baseline (time 1) to 6 months (time 3). *Post hoc* analysis revealed no significant change in self-management and transition readiness from time 2 (immediately after the intervention) to time 3 (6 months). Overall, the effects of remaining outcomes were also not significant (see Table 3).

**Table 3:** Effects of the iPeer2Peer congenital heart disease program on secondary effectiveness outcomes for mentees

Outcome	Time 1 vs time 2, β (95% CI)	*P* value	Time 1 vs time 3, β (95% CI)	*P* value	Time 2 vs time 3, β (95% CI)	*P* value
Self-management and transition readiness	1.81 (–2.71, 6.33)	0.433	**6.37(1.97, 10.7)**	**0.005**	4.561757 (–0.11, 9.23)	0.056
PedsQL cardiac module	1.36 (–6.89, 9.61)	0.746	–0.25 (–8.50, 7.99)	0.952	–1.61 (–10.2, 6.96)	0.712
PedsQL treatment	–6.43 (–11.1, –1.81)	0.006	–2.55 (–7.51, 2.40)	0.313	3.88 (–1.37, 9.13)	0.148
PedsQL appearance	–0.66 (–9.22, 7.89)	0.879	0.50 (–8.05, 9.05)	0.909	1.16 (–7.70, 10.0)	0.797
PedsQL treatment anxiety∗	3.68 (–5.28, 12.7)	0.421	0.0016545 (–8.97, 8.97)	1.000	–3.68 (–12.9, 5.60)	0.437
PedsQL cognitive	–0.82 (–10.2, 8.56)	0.864	–2.14 (–11.5, 7.23)	0.654	–1.32 (–11.1, 8.42)	0.790
PedsQL communication	–1.20 (–9.49, 5.52)	0.604	2.93 (–4.57, 10.4)	0.44	4.91 (–2.85, 12.7)	0.215
Cardiac knowledge	2.11 (–1.10, 5.32)	0.198	0.76 (–2.45, 3.97)	0.644	–1.35 (–4.66, 1.95)	0.422
Peer relationships	–0.21 (–4.75, 4.34)	0.929	–1.87 (–6.29, 2.56)	0.409	–1.66 (–6.39, 3.06)	0.492
Self-efficacy	1.13 (–3.24, 5.51)	0.612	–0.75 (–5.13, 3.62)	0.736	–1.89 (–6.46, 2.69)	0.419

Statistically significant values are indicated in bold.

CI, confidence interval; PedsQL, Pediatric Quality of Life Inventory.

∗ This question was only applicable to participants currently taking heart medication; therefore, the sample size for this outcome was n = 7.

This finding was supported by the qualitative data analysis, which demonstrated that a major concern for youth and young adults was navigating the transition to adulthood. In semistructured interviews, mentees reported feelings of anxiety and uncertainty about transitioning to new health care and educational systems as one of the motivating factors for enrolling in the peer mentorship program.

There was a lot of fear changing their cardiologist, as well as their healthcare team... to go from a children’s hospital where… you’re used to being around kids, to an adult hospital where you’re […] seeing people from 18 to 100. So it's a bit of a change for sure. (24-year-old woman mentor)

Many shared that speaking with peer mentors brought a sense of reassurance and allowed them to appreciate their own capabilities. Mentees found that their mentor’s positive outlook on their own diagnoses encouraged them to adopt a more optimistic mindset.

I learned, you know, that I can still do things that other people do. I’m a fully capable person, I just need to kind of be mindful of my own limits... it’s part of who you are but it’s not—like it doesn’t define you. (16-year-old mentee)

By sharing insights on navigating adult health care systems and discussing the differences they had encountered, mentors helped prepare mentees for the transition to adult care:

I shared my experience of getting a catheterization last year as well as going through some medication changes, and […] moving on to adult care... it eased some fears, and by the end of it, they weren’t so scared about going into [adult hospital]. (24-year-old woman mentor)

## Discussion

This study aimed to investigate the feasibility and acceptability of a virtual peer support mentorship program for AWCHD alongside a clinical transition program. The quantitative results demonstrated high levels of engagement and program adherence and significant improvements in adolescents’ transition readiness over time. The qualitative findings revealed strong support and acceptability within this population for mentees, mentors, and HCPs. This study highlights the role of peer mentorship as a unique form of psychosocial support for youth and young adults as they navigate the transition into adulthood and the adult health care system.

Notably, a recent priority-setting study conducted by Keir et al.^41^ identified peer support interventions as one of the top 10 current research priorities for teen and adult patients with CHD. In their study, teenage patients reported being especially concerned with “fitting in” and discussed experiencing secrecy and embarrassment about living with CHD.^41^ Patients also discussed the mental health challenges they experienced, and teens emphasized their anxiety surrounding the transition process in particular.^41^ These findings align with the results of this study, where patients most often cited nervousness about transitioning as a motivator to enroll in the program, and the significant improvements in transition readiness scores 6 months after the program demonstrate the ability of peer mentorship programs to address these concerns in AWCHD.

In the present study, AWCHD reported positive program experiences and highlighted the unique role this program filled in connecting them with young adults who also lived with CHD and had successfully transferred to adult care. This research is the first to highlight the utility of peer support programs for AWCHD, and these encouraging results indicate the need to continue evaluating and improving the psychosocial support for this population. This study also demonstrated that a virtual peer mentoring program can be implemented alongside existing clinical transition programs as an additional source of support during the transition process.

Current transition guidelines suggest beginning the transition education and process at 12 years old.^11^ In practice, the results of our study suggest that AWCHD were not actively engaged in transition planning at this stage and were often not interested in peer mentorship surrounding this process until closer to 16 years of age. This may also be related to challenges with cognitive functioning observed in youth with more severe CHD, which can limit their engagement in transition planning earlier in the trajectory.^42^ Often parents are engaged in planning the transition to adult care, and therefore, it is important to capitalize on parent-targeted interventions. Previous research has demonstrated a need for adequate education and preparation to allow parents to feel more secure in handing off the responsibility of their child’s health care management.^43^ Specifically for AWCHD, a recent qualitative study revealed that both patients and parents would feel more prepared for the transition to adult care if adolescents had opportunities to improve transitional skills and increase their knowledge related to their CHD diagnosis and treatment.^44^ There is also evidence to suggest that a peer support intervention can provide parents with emotional relief and creates a platform to learn from other parent’s experiences in an open conversation.^45^ This is further supported by findings that autonomy in children with chronic diseases tends to emerge later in life, with parent-dependent relationships tending to last longer.^46^

A unique feature of this iPeer2Peer CHD program was the high number of men enrolled, as several previous iPeer2Peer trials demonstrated enrollment of predominantly women participants.^24^, ^25^, ^26^ Specifically, a pilot RCT of the iPeer2Peer program in adolescents with juvenile idiopathic arthritis contained a sample comprising 97% women participants.^26^ In contrast to other studies that often match predominantly on gender, matching in this program prioritized disease severity and other social factors, which allowed for cross-gender pairings (when participants were willing and open to being matched with a mentor of different gender). In fact, most mentees who were men had no preference for a mentor who was a man vs a woman. These findings further highlight the interest in and acceptability of a peer support program across genders in the AWCHD population.

Finally, the AWCHD group was highly engaged in the iPeer2Peer program. With the average length of calls being 40 minutes and some dyads speaking for as long as 70 minutes, there was a clear role for mentors to provide unique social support. Compared with previously published iPeer2Peer trials in other disease populations that report median call lengths of 20 minutes, it seems that AWCHD were particularly interested in speaking to their mentors for longer periods of time.^24^ In addition to addressing the well-documented experiences of social exclusion and stigma for youth with CHD, there was an urgent need to implement this virtual support intervention as the COVID-19 pandemic and associated restrictions limited opportunities for AWCHD to receive typical forms of social support.^3^^,^^47^

### Limitations

Despite the successful outcomes demonstrated in this study, there are several limitations to this research. First, this study used a pretest posttest design; therefore, there was no control group with which to compare outcomes to determine effectiveness of the program. Future research could engage a RCT design to establish program effectiveness on psychosocial outcomes. This study was also embedded within a new clinical program (SickKids Cardiac Transition Program), which impacted the initial rate of recruitment and enrollment. However, this was addressed by expanding recruitment sites into the general CHD clinic follow-up visits to increase enrollment. Despite expanding recruitment sites, our enrollment rate only reached 25%. This enrollment rate is likely multifactorial; some potential participants stated that they did not feel the program was necessary for them (especially those in the younger age range who were not yet preparing for transition, as well as those with minor lesions or less severe CHD), and many were busy with final examinations and extracurricular activities and stated that they did not have sufficient time to participate. Because this study required completion of a consent call, survey measures at 3 time points, and mentorship calls, it may have been perceived as time-consuming for some patients. To address this, future research may limit enrollment to those with moderate to severe disease and shift the target age range to mentees who fall into an older age range (ie, 16-21 years old). If implemented beyond the research setting, the time burden for participants may significantly decrease as activities such as consent calls, time point measures, and call monitoring may not be required. Finally, it was difficult to maintain engaged mentors early in the program when recruitment was slow, which led to 2 mentors dropping out. Likewise, it was also challenging for the program to handle fluctuations in interest when enrollment increased due to limited mentor capacity. This meant that a few participants experienced a time lag between enrollment and being matched with a mentor to complete the calls. Future research may consider increasing the number of mentors to account for fluctuations in availability and enrollment rates. However, a strength of this study was the diversity of mentors (range of disease severity, ethnicity, age, interests, and genders), which allowed for high-quality matching of dyads.

## Conclusion and Future Directions

The iPeer2Peer CHD program is one of the first virtual peer mentorship programs to be developed and implemented for youth with CHD to guide their transition to adult care. This study demonstrated that there is sufficient interest in a subset of AWCHD, as well as a need for this type of program to support adolescents as they navigate the transition to adulthood and into the adult care setting. This program was acceptable to both mentees and mentors, as well as HCPs who integrated referrals to the program into the clinical flow of visits within the clinical setting. Pediatric and adult CHD clinical programs may consider implementing similarly structured peer support initiatives, as this program demonstrated improvements in transition readiness for mentees who participated. This finding also aligned with qualitative data that highlighted the benefits of this program as an additional support during the often stressful and overwhelming transition process.

Future directions for this research program may include a national, multisite pilot RCT to demonstrate feasibility and preliminary effectiveness of outcomes. Based on the results of this program that found more interest among older mentees and those with more severe CHD, future peer mentorship programs may consider focusing on slightly older demographic of mentees (ie, starting at age 16 years) and targeting a population of moderate to severe CHD who may benefit most from this type of support. In addition, incorporating support programs for parents of youth who are preparing for transition would be beneficial.
