# Supplementary material for: Virtual Peer Mentoring for Adolescents With Congenital Heart Disease: A Mixed-Methods Study of the iPeer2Peer Program in the Transition to Adult Care
Source: CJC Pediatr Congenit Heart Dis. 2025 Apr 17;4(6):295–305. doi: 10.1016/j.cjcpc.2025.04.003 (PMC12835979; doi:10.1016/j.cjcpc.2025.04.003)
Supplement: Supplemental Table S1 [file mmc1.pdf]

Supplemental Tables

Supplemental Table S1. Study questionnaire completion by time point.

	Time 1 – Baseline n(%)	Time 2 – Post iPeer2Peer program n(%)	Time 3 – 6 Months n(%)
**MENTEE (n=18)**			
TRANSITION-Q	18 (100%)	13 (72%)	14 (77%)
PedsQL Cardiac Module Version	18 (100%)	14 (77%)	14 (77%)
SickKids CHD Knowledge Questionnaire	18 (100%)	14 (77%)	14 (77%)
Pain Questionnaire	18 (100%)	14 (77%)	14 (77%)
PROMIS Pediatric Peer Relationships	18 (100%)	13 (72%)	14 (77%)
Sherer's Generalized Self-Efficacy Scale	18 (100%)	14 (77%)	14 (77%)
Mentor Behavior Scale	NA	14 (77%)	NA
Peer Mentor Support Scale	NA	14 (77%)	NA
**MENTOR (n=7)**			
	Time 1 – Baseline n(%)		Time 2 – Post iPeer2Peer n(%)
36-Item Short Form Health Survey (SF-36)	7 (100%)		5 (72%)
PROMIS Satisfaction with Social Roles and Activities	7 (100%)		5 (72%)

Supplemental Table S2. Effects of the iPeer2Peer Congenital Heart Disease program on secondary effectiveness outcomes for mentors

	Time 1 – Baseline n=7	Time 2 – Post iPeer2Peer n=5
36-Item Short Form Health Survey (SF-36)*		
Physical functioning, median(IQR)	100 (90 - 100)	95 (85 - 95)
Role limitations due to physical health, median(IQR)	100 (50 - 100)	100 (75 - 100)
Role limitations due to emotional problems, median(IQR)	100 (100 - 100)	100 (0 - 100)
Energy/fatigue, mean(SD)	60.0 (25.3)	51.0 (12.9)
Emotional well-being, mean(SD)	65.5 (16.9)	54.4 (12.8)
Social functioning, mean(SD)	75.0 (23.9)	65.0 (41.8)
Pain, mean(SD)	79.6 (17.5)	82.5 (22.9)

General Health, mean(SD)	42.0 (19.5)	42.6 (9.12)
PROMIS Satisfaction with Social Roles and Activities**	52.3 (10.2)	45.7 (13.7)

* Higher score define a more favorable health state ** A higher PROMIS T-score indicates a greater level of satisfaction.
